# The jumping mechanism of flea beetles (Coleoptera, Chrysomelidae, Alticini), its application to bionics and preliminary design for a robotic jumping leg

**DOI:** 10.3897/zookeys.915.38348

**Published:** 2020-02-24

**Authors:** Yongying Ruan, Alexander S. Konstantinov, Guanya Shi, Yi Tao, You Li, Andrew J. Johnson, Xiaozhu Luo, Xinying Zhang, Mengna Zhang, Jianing Wu, Wenzhu Li, Siqin Ge, Xingke Yang

**Affiliations:** 1 Key Laboratory of Zoological Systematics and Evolution, Institute of Zoology, Chinese Academy of Sciences, Beijing, China; 2 School of Applied Chemistry and Biological Technology, Shenzhen Polytechnic, Shenzhen, Guangdong 518055, China; 3 Systematic Entomology Laboratory, USDA, ARS, c/o Smithsonian Institution, National Museum of Natural History, Washington DC, USA; 4 Department of Automotive Engineering, Tsinghua University, Beijing, China; 5 Key Lab of Animal Ecology and Conservation Biology, Centre for Computational Biology and Evolution, Institute of Zoology, Chinese Academy of Sciences, Beijing, China; 6 School of Forest Resources and Conservation, University of Florida, Gainesville, FL, USA; 7 Department of Entomology, South China Agricultural University, Guangzhou, China; 8 School of Aeronautics and Astronautics, Sun Yat-Sen University, Guangzhou, 510006, China

**Keywords:** bionics, catapult, functional morphology, jump, kinematics, metafemoral spring, robotics

## Abstract

Flea beetles (Coleoptera, Chrysomelidae, Galerucinae, Alticini) are a hyperdiverse group of organisms with approximately 9900 species worldwide. In addition to walking as most insects do, nearly all the species of flea beetles have an ability to jump and this ability is commonly understood as one of the key adaptations responsible for its diversity. Our investigation of flea beetle jumping is based on high-speed filming, micro-CT scans and 3D reconstructions, and provides a mechanical description of the jump. We reveal that the flea beetle jumping mechanism is a catapult in nature and is enabled by a small structure in the hind femur called an ‘elastic plate’ which powers the explosive jump and protects other structures from potential injury. The explosive catapult jump of flea beetles involves a unique ‘high-efficiency mechanism’ and ‘positive feedback mechanism’. As this catapult mechanism could inspire the design of bionic jumping limbs, we provide a preliminary design for a robotic jumping leg, which could be a resource for the bionics industry.

## Introduction

There are as many as 380,500 described species of recent beetles (Insecta, Coleoptera) in the world (Slipinski et al. 2011). Apart from walking, running and flying, beetles have developed various other types of locomotion with a main purpose to escape predators, such as clicking (e.g., Elateridae), tumbling (e.g., Mordellidae) and jumping (e.g., Chrysomelidae, Alticini; Scirtidae; Curculionidae, Ceutorhynchinae; and Rhamphini).

Apart from jumping beetles, some other arthropods are also well known for having rapid-moving appendages, for example: jumping legs in fleas (Bennet-Clark and Lucey 1967), locusts (Bennet-Clark 1975), froghoppers (Burrows 2006) and leafhoppers (Bonsignori et al. 2013); power-amplified mandibles in trap-jaw ants (Patek et al. 2006; Larabee et al. 2017); snapping mandibles in termite soldiers (Seid et al. 2008); raptorial appendages in mantis shrimp (Versluis et al. 2000; Lohse et al. 2001; Patek et al. 2004, 2013); snapping chelicerae in trap-jaw spiders (Wood et al. 2016); etc. The snapping movement of the rapid-moving appendages are usually driven by the storage and rapid release of elastic potential energy (Patek et al. 2013), serving to the conservation of metabolic energy and the amplification of muscle power output, which allows the locomotor system to operate beyond the bounds of intrinsic muscle properties (Roberts and Azizi 2011).

The extraordinary jumping ability of flea beetles mainly depends on the metafemoral spring (Furth 1988) in the dilated femur of their hind legs, which enables them to perform the catapult jump. The jumping of flea beetles is an extremely effective method to avoid potential predators, as it allows beetles quickly disappear from the leaf surface, where they spend most of their life. *Blepharidasacra* (Weise) can jump up to 70 cm or 100 times more than its body length (Furth 1988), while *Longitarsusanchusae* (Paykull) reaches a jump of 289 times its body length (Schmitt 2004); the average acceleration of *Psylliodesaffinis* (Paykull) during take-off can be up to 266 times the acceleration of gravity (Brackenbury and Wang 1995).

Maulik (1929) was the first to document the internal ‘springs’ (metafemoral spring) inside the hind femur of flea beetles. Not long after the metafemoral spring was described, Lever (1930) discovered another internal structure inside the flea beetle’s hind femur: Lever’s triangular plate. Since then, numerous studies have been conducted in many aspects of flea beetle’s metafemoral spring, such as morphology (Furth 1980, 1982; Furth et al. 1983; Furth and Suzuki 1998; Lingafelter and Konstantinov 1998; Schmitt 2004), behavioristics (Brackenbury and Wang 1995), systematics (Furth 1985, 1989; Furth and Suzuki 1994) and evolution (Furth 1992; Ge et al. 2011). Barth (1954) attempted to explain the mechanism of the flea beetle’s jump. He studied the musculature of the hind femur of *Omophoitasexnotata* (Harold) and suggested that both metafemoral spring and Lever’s triangular plate are involved in the jumping, functioning similar to the mechanism of a catapult. Barth based this conclusion solely on the anatomical data from a single species. His theory was subsequently questioned by Furth (1980, 1988), who believed that more details and structures should be studied, and the true function of this jumping mechanism remains a mystery in his opinion (Furth 1982). Nadein and Betz (2016) published the 3-D structure of the flea beetle hind leg, and studied the performance of several flea beetle species, their study suggested a different theory of jumping mechanism from that proposed by Barth (1954).

In order to gain comprehensive insights into the mechanics behind the flea beetle jump, we conducted micro-CT scans, 3D reconstructions, high-speed filming and dissection of the metafemur. As a result, the ‘elastic plate’ and its function in the hind legs of flea beetles is revealed; a comprehensive theory of the mechanism involved in flea beetle jumping is given. In addition, and based on our findings, we provide a design diagram for a robotic jumping leg.

## Material and methods

### Micro-CT scanning analysis

Absolute ethanol-preserved specimens of flea beetles were selected. The meta-femurs were carefully removed and dried at the critical point (hcp-2, Hitachi Inc., Tokyo, Japan), and then glued to the tip of a micropipette using nail polish. Hind legs of seven species [*Alticacirsicola* Ohno, *Clavicornaltica* sp., *Hesperalomasa* Maulik, *Nonarthra* sp., *Asiophridaxanthospilota* Baly, *Podontialutea* (Olivier, 1790), *Psylliodes* sp.] were scanned with a MicroXCT-400 scanner (Xradia Inc., California, USA. Beam strength: 60 kV, absorption contrast). Pixel size of images: 0.5~5μm; optical magnification: 4–40× (depending on different specimen size). In most scans, 900–1100 sections of images were obtained, then imported to Amira 5.4.1 (Visage Imaging, San Diego, California, USA) for 3D reconstructions. Autodesk maya 2014 (Autodesk Inc., San Rafael, California, USA) was used to smooth and render the 3D structures.

Hind legs of flea beetles across 13 genera were further dissected and examined (list of species dissected: *Agasicleshygrophila* Selmen et Vogt, *Alticacirsicola*, *Chaetocnemaconstricta* Ruan, Konstantinov et Yang, *Clavicornaltica* sp., *Hemipyxis* sp., *Hesperalomasa*, *Luperomorphaxanthodera* (Fairmaire), *Nonarthra* sp., *Asiophridaxanthospilota*, *Podontialutea*, *Psylliodes* sp., *Stenoluperus* sp., *Trachytetraobscura* (Jacoby)). These genera and species were chosen to represent flea beetles with different body sizes. A conventional optical imaging system consisting of a Zeiss Axiostar plus microscope (Zeiss Inc., Göttingen, Germany), Nikon D300 digital camera (Nikon Inc., Tokyo, Japan) and Helicon Focus 6 software was used to capture and compose 2D images. The figure plates were prepared with Photoshop CS5 (Adobe, San Jose, USA) and Illustrator CS5 (Adobe, San Jose, USA).

The general morphological terminology used throughout this report follows Furth (1988).

### High-speed filming

Four species of flea beetles (*Chaetocnemapicipes* Stephens, *Alticacirsicola*, *Asiophridaxanthospilota*, *Psylliodespunctifrons* Baly) were collected in the field in Beijing, China from July to October 2015 for high-speed filming. During the study, the flea beetles were reared in the laboratory in plastic containers and fed on their host plants. Videos of their jumps were recorded at 4580–6800 fps using a Phantom M110 high-speed camera (Vision Research Inc., USA). Take-off velocity and acceleration were determined by the recorded videos, which were played frame-by-frame and analyzed using PCC 2.5 software (PHANTOM CAMERA CONTROL 2.5.744.0, Vision Research Inc., USA).

## Results

### Morphology of the flea beetle hind leg (Figs 1–3)

**Figure 1.**
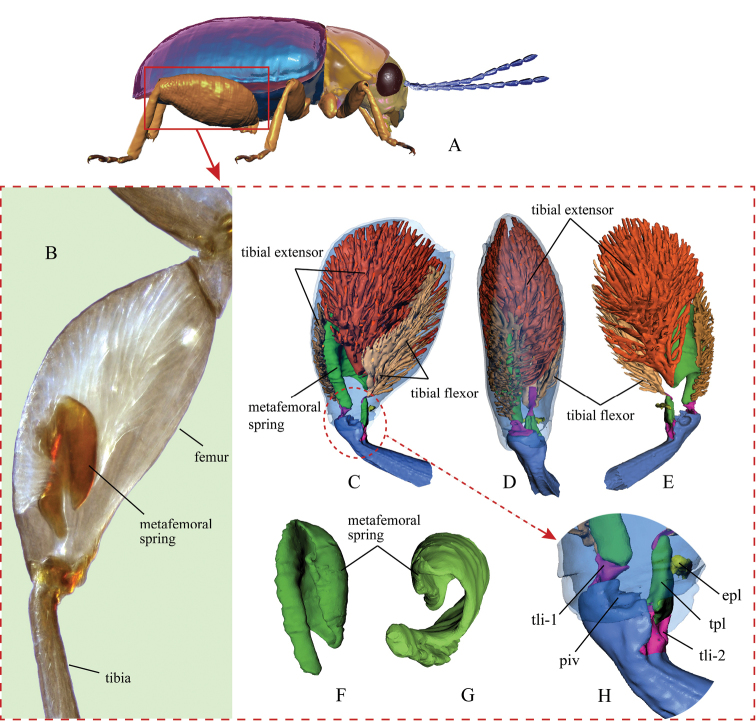
Jumping apparatus in the flea beetle hind leg. **A** a model of a generic flea beetle (lateral view), indicating the enlarged hind leg **B** hind leg of *Trachytetraobscura* under a light microscope (dark-field microscopy) **C–H** X-ray computer tomography-based 3D reconstructions of the hind leg of the flea beetle *Asiophridaxanthospilota***C** lateral view of the hind leg and internal structures **D** dorsal view of the hind leg **E** lateral view of the hind leg (view from an opposite direction of inset C) **F** lateral view of the metafemoral spring **G** ventral view of the metafemoral spring **H** femorotibial joint. Abbreviations: ***epl***: elastic plate; ***piv***: tibial pivot of femorotibial joint; ***tli-1***: primary tibial ligament; ***tli-2***: secondary tibial ligament; ***tpl***: Lever’s triangular plate.

Our findings on the hind leg musculature are mostly in accord with those described by Barth (1954) and Nadein and Betz (2016). However, our 3D reconstructions and dissection show that flea beetles have another small structure related to jumping in their hind leg, described here as the ‘elastic plate’ (Figs 1H, 5F: ***epl***), it resembles a small tendon: semi-transparent, milky white, nearly ellipsoid and elastic structure with weak sclerotization; it is attached to the inner wall of the femur, ventro-distally, situated near the femorotibial joint (Fig. 5H). The base of the elastic plate is sclerotized and fused with the inner wall of femur, while the apical and middle part is rubber-like and has great elasticity.

**Figure 2.**
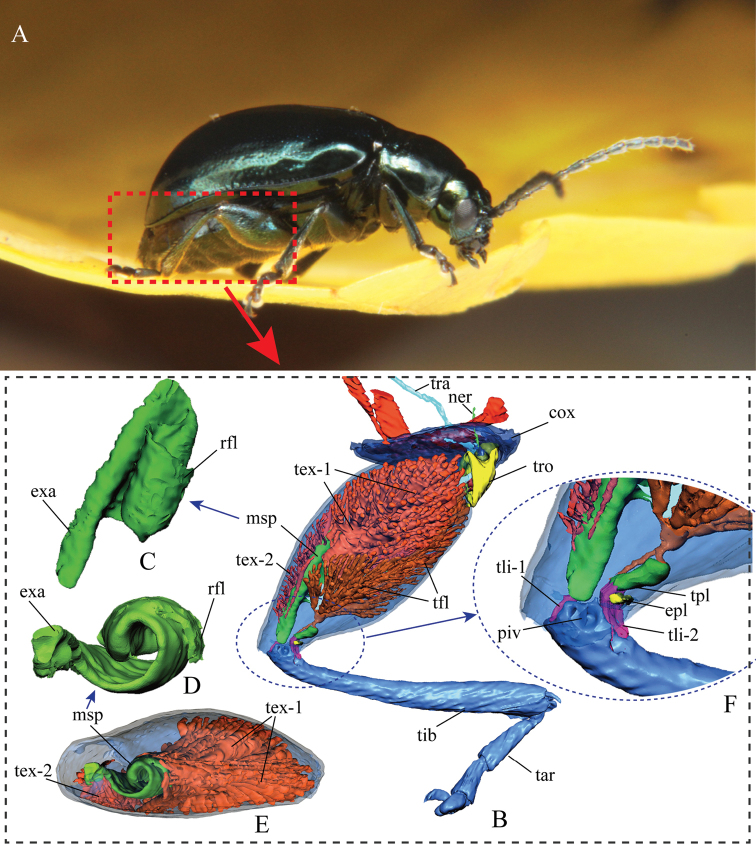
Internal structures of the hind leg of *Alticacirsicola*. **A** the flea beetle *Altica* sp. on foliage **B–F** X-ray computer tomography 3D reconstructions of the hind leg of *A.cirsicola***B** lateral view of the left hind leg **C** lateral view of the metafemoral spring **D** distal view of the metafemoral spring **E** distal view of the metafemoral spring and tibial extensors **F** features of the femorotibial joint. Abbreviations: ***cox***: coxa; ***exa***: extended arm of the metafemoral spring; ***epl***: elastic plate; ***fem***: femur; ***msp***: metafemoral spring; ***ner***: nerve; ***piv***: tibial pivot of the femorotibial joint; ***rfl***: recurve flange of the metafemoral spring; ***tar***: tarsi; ***tex-1***: primary tibial extensor; ***tex-2***: secondary tibial extensor; ***tfl***: tibial flexor; ***tib***: tibia; ***tli-1***: primary tibial ligament; ***tli-2***: secondary tibial ligament; ***tpl***: Lever’s triangular plate; ***tra***: trachea; ***tro***: trochanter.

**Figure 3.**
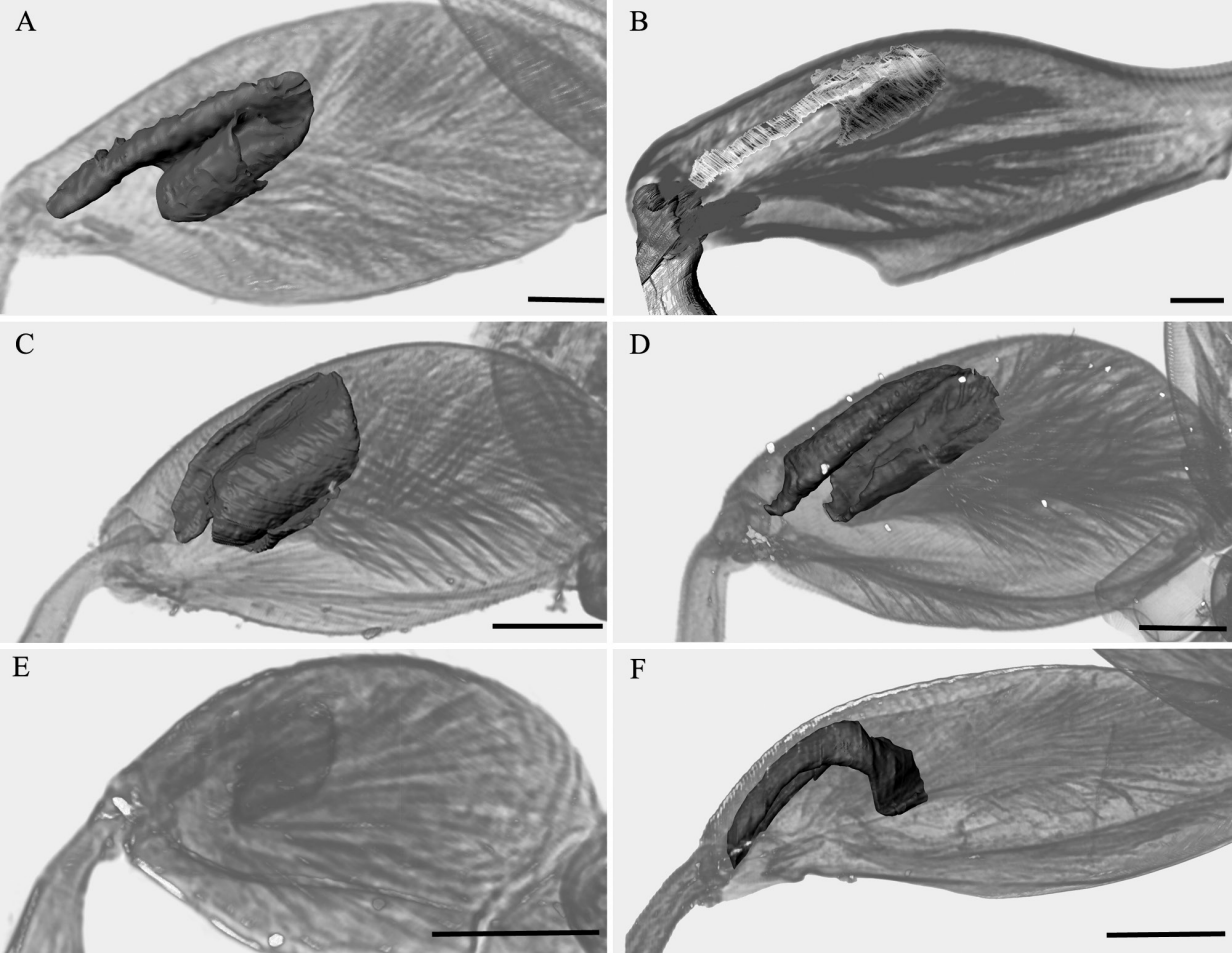
Variation in the metafemoral spring of different flea beetle species (3D reconstructions). **A** hind femur of *Alticacirsicola***B** hind femur of *Podontialutea***C** hind femur of *Psylliodes* sp. **D** hind femur of *Nonarthra* sp. **E** hind femur of *Clavicornaltica* sp. **F** hind femur of *Hesperalomasa*. Scale bar: 0.2 mm.

The elastic plate was found inside the hind femora of all 13 genera of flea beetles that were dissected. In some other jumping insects that we have examined [Galerucines (Chrysomelidae, Galerucini); *Scirtes* sp. (Coleoptera, Scirtidae); *Rhynchaenus* sp. (Coleoptera, Curculionidae); *Lycormadelicatula* (White) (Hemiptera, Fulgoridae); *Locustamigratoria* (L.) (Orthoptera, Acrididae); Tridactylidae sp. (Orthoptera, Tridactylidae); *Brachymeria* sp. (Hymenoptera, Chalcididae)], the elastic plate is absent. However, a thin membrane is present in the same area, which may serve to protect the femorotibial joint in locomotion. It is very possible that the elastic plate is developed from this membrane.

### The flea beetle jumping process (Fig. 4)

**Figure 4.**
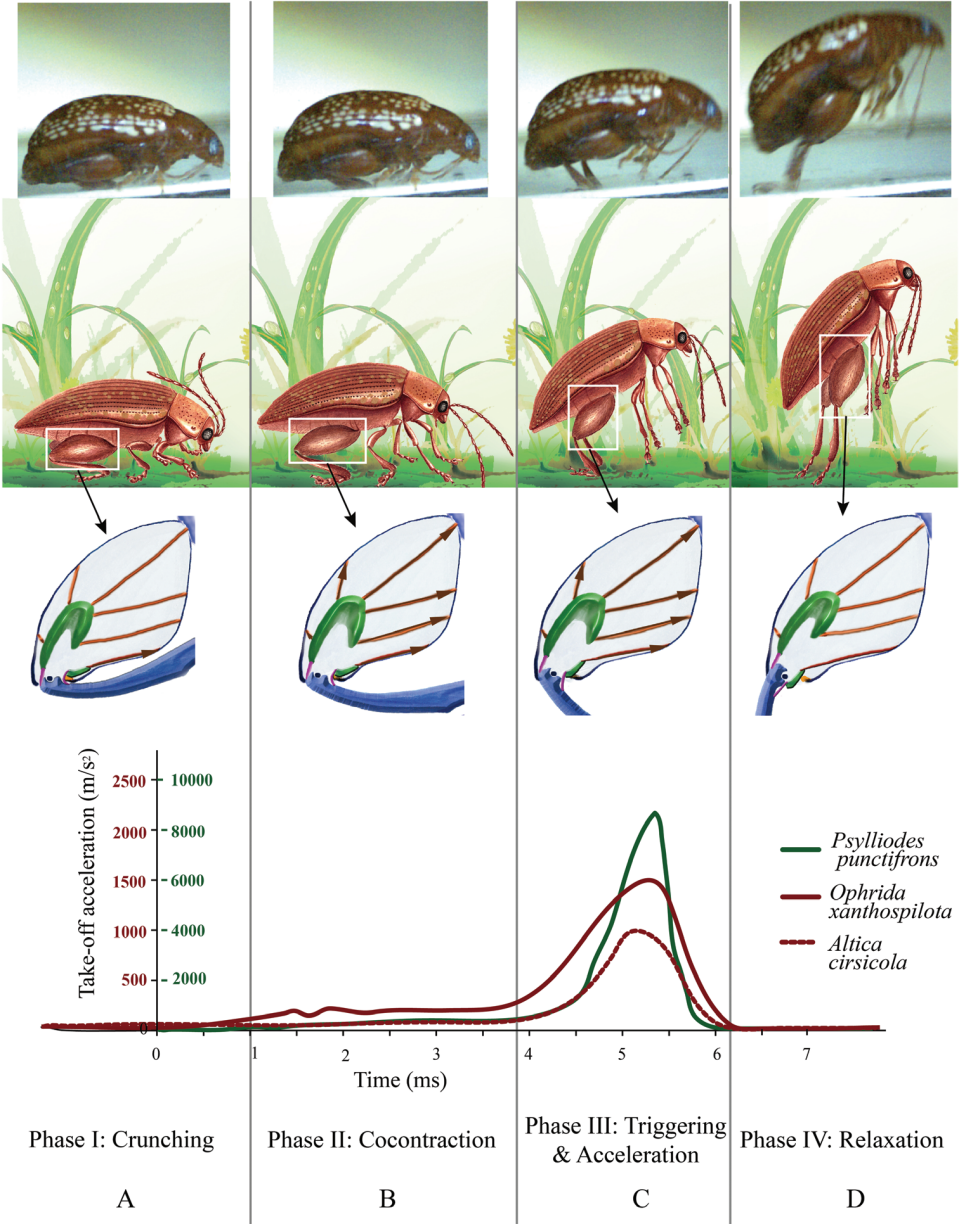
Take-off strategy of flea beetles (*Asiophridaxanthospilota* is shown in the photos at the top of the figure). Acceleration data were calculated based on three typical jumps recorded by a high-speed camera. The three different species were chosen to represent flea beetles with different sizes **A** Phase I (Crunching): tibial flexor muscles contract, causing flexion of the tibia **B** Phase II (Co-contraction): tibial extensor muscles and tibial flexor muscles contract simultaneously, catching the triangular plate and hindering the extension of the tibia **C** Phase III (Triggering and Acceleration), the triangular plate is dislodged, causing the explosive release of energy **D** Phase IV (Relaxation), the flea beetle is catapulted into the air and the muscles begin to relax.

Based on our morphological observations and the high-speed filming, we hypothesize that a typical flea beetle jump can be divided into four major phases (Fig. 4, Supplementary files movie S1–S3).

Phase I entails the preparation for a jump, wherein the flea beetle flexes its hind legs until the femorotibial angle reaches a minimum of approximately 20°. This flexion usually takes no more than 20 ms (Fig. 4A).

**Figure 5.**
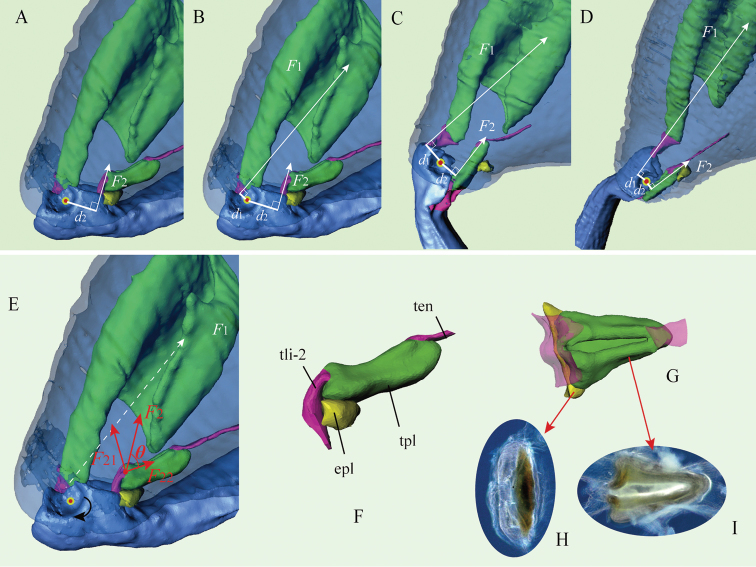
The dynamics of the catapult mechanism of the flea beetle hind leg powered by the elastic plate (*Asiophridaxanthospilota*, micro-CT 3D reconstructions from four different phases of the jumping leg). (*F*_1_ indicates the force generated by tibial extensor muscles responsible for extension of the tibia; *F*_2_ indicates the force responsible for flexion of the tibia which is indirectly generated by tibial flexor muscles). **A–D** 3D reconstructions of four hind legs through each of the four phases of the jump, indicating variable *d*_1_ (moment arm of *F*_1_) and *d*_2_ (moment arm of *F*_2_). *d*_1_ is at its minimal length at the start of the hind leg extension and at its maximal length during the middle; by contrast, *d*_2_ is at its maximal length at the start of the hind leg extension and at its minimal at the end **A** phase I **B** phase II **C** phase III **D** phase IV **E** the positive feedback mechanism in the take-off process. *F*_2_ comprises *F*_21_, which is the constrained force generated by the elastic plate, and *F*_22_, which is generated by the tibial flexor muscle **F** the triangular plate – elastic plate complex (lateral view) **G** the triangular plate – elastic plate complex (dorsal view) **H** the elastic plate (dorsal view, under light microscope, dark-field microscopy) **I** the triangular plate (dorsal view, under light microscope, dark-field microscopy). Abbreviations: ***epl***: elastic plate; ***tli-2***: secondary tibial ligament; ***tpl***: triangular plate; ***ten***: tendon of tibial flexor.

Phase II is an initiation phase in which elastic strain energy cumulatively builds up inside the femur as the femorotibial angle increases from approximately 20° to 60°, which takes approximately 4–5 ms (Fig. 4B). Inside the femur, the tibial extensor and tibial flexor muscles contract simultaneously [Co-contraction (Nadein and Betz 2016)]. Given that the tibial extensor muscle generates greater force than the tibial flexor, the tibia starts to revolve (or extend) around the tibial pivot (Fig. 1H: ***piv***). As a result, the triangular plate (also as: Lever's triangular plate) (Fig. 5F: ***tpl***) could be drawn out of the femur, but momentarily gets caught by and stuck on the elastic plate (Fig. 5F: ***epl***). Thus, the metafemoral spring (Figs 1F, G; 3) is stretched by the tibial extensor muscle, establishing a catapult-like structure inside the femur. The metafemoral spring stores the huge elastic strain energy required to trigger the catapult, while the triangular and elastic plates comprise a ‘trigger system’ that catches the ‘sling’ and prepares to trigger the catapult.

Phase III is the most dramatic phase, yet it only takes 1–2 ms (Fig. 4C). Due to an increase in the femorotibial angle and accumulating tension, the elastic plate is no longer able to hold the catapult sling in position; subsequently, the triangular plate is dislodged abruptly from the elastic plate and slips out of the femur, triggering the explosive jump. The acceleration of the flea beetle increases explosively, peaking in less than 1 ms, and then abruptly dropping to near zero. The femorotibial angle extends from approximately 60° to 130° in 1–2 ms.

Finally, in phase IV (Fig. 4D), when the femorotibial angle reaches approximately 130°, the acceleration drops to almost zero, and no more strain energy is released. After the femorotibial angle extends beyond approximately 160°, the flea beetle leaves the ground.

Furthermore, an experiment was designed to test the four phases of the catapult mechanism identified by the position of the elastic plate during a simulated jump (Supplementary files movie S4).

### Mechanical analysis of the flea beetle jump – the ‘Positive Feedback Mechanism’ (Fig. 5)

To understand the explosive manner of the flea beetle jump, we analyzed the mechanical dynamics involved in the jumping process.

For the mechanical analysis, we generated 3D reconstructions of the hind legs during the four different phases of the catapult jump (Fig. 5). Here we use *F*_1_ and *F*_2_ to denote two forces acting on the tibia via the tibial ligaments, where *F*_1_ is generated by the tibial extensor muscle and *F*_2_ is indirectly generated by the tibial flexor muscle; *d*_1_ and *d*_2_ denote the moment arms (i.e., lever arms) of *F*_1_ and *F*_2_, respectively, corresponding to the femorotibial joint. The total torque generated by muscle around the joint (i.e., the tibial pivot), denoted by *M*, can be expressed as *M=F*_1_*d*_1_–*F*_2_*d*_2_. The 3D reconstructions of the hind leg at phases I, II, III and IV of the jump are detailed in panels A, B, C and D of Fig. 5, respectively, which shows how the force *F_j_* and its moment arm *d_j_* (*j*=1, 2) change during the different phases. During the extension of the hind leg, *d*_1_ is at its minimum at the start of the extension and at its maximum during the middle of the extension; by contrast, *d*_2_ is at its maximum at the start of the extension and at its minimum at the end. Furthermore, the force *F*_2_ comprises two forces, *F*_21_ and *F*_22_, where *F*_21_ is the constrained force generated by the elastic plate and *F*_22_ is generated by the tibial flexor muscle (Fig. 5E). *F*_21_ and *F*_22_ can be simply expressed as *F*_21_=*F*_2_*sin*θ and *F*_22_=*F*_2_*cos*θ, where θ is the angle between *F*_2_ and *F*_22_ (Fig. 5E). The lateral and dorsal views of the triangular plate – elastic plate complex are shown in Fig. 5F, G, and the dorsal views of the elastic plate and triangular plate under a light microscope are shown in Fig. 5H, I.

At the beginning of phase II, the total torque *M* is close to zero, since *F*_1_*d*_1_≈*F*_2_*d*_2_. Therefore, in this phase, the tibia extends very slowly, and the metafemoral spring is stretched by *F*_1_ and *F*_22_, such that the huge energy generated by the antagonistic muscles (tibial extensor and flexor) is stored. In phase III, the continuous extension of the hind leg causes a rapid increase in *d*_1_ while *d*_2_ decreases; simultaneously, θ decreases and *F*_22_ does not change, thereby dramatically decreasing *F*_2_=*F*_22_/*cos*θ. This ‘positive feedback mechanism’ leads to the explosive increase in the total torque *M=F*_1_*d*_1_–*F*_2_*d*_2_ (at this point, *F_1_d_1_*>>*F_2_d_2_*). When θ decreases to near zero, the triangular plate is dislodged and slips out of the femur. Meanwhile, the enormous elastic strain energy, previously stored in the metafemoral spring, is rapidly converted into kinetic energy, allowing the flea beetle to attain an extraordinarily high acceleration.

Taken together, these results suggest that two structures play a significant role in the catapult mechanism – the elastic plate and the triangular plate, which control the timing of the jumping process, the triggering of the catapult, and the explosive release of energy.

### ‘High Efficiency Mechanism’ of the flea beetle jump

In order to explore the efficiency of flea beetle jumping, we compared their jumping dynamics with that of humans. Morphologically, the flea beetle hind leg resembles the human leg since both are ‘two-segment systems’ (Alexander 1995). In a typical human jump, assuming the tibial extensor muscle generates a constant force, the uplift force and work efficiency of the leg are almost proportional to the knee angle (femorotibial angle) (Martin and Stull 1969; Zhao and Li 2008). The dynamics of this mechanism can be generally expressed as: *F* α *tan β* (where *F* is the uplift force and *β* is the knee angle) (Zhao and Li 2008), which means that the energy generated by the tibial extensor muscle (or the elastic strain energy stored in elastic elements) can be turned into kinetic energy more efficiently at a later stage of a jump. Similarly, instead of accelerating constantly throughout its jump, the flea beetle also uses this high-efficiency mechanism to store colossal strain energy at an early stage and release it at a later, higher-efficiency stage. In this way, the flea beetles avoid muscle fatigue (or energy waste) and improve their jumping performance simultaneously.

High-speed film data demonstrate that the peak acceleration is approximately 10 times as great as that at the start of the jump (in Phase II). As shown by our kinematic data based on high-speed filming, *Psylliodespunctifrons* jumped with an average acceleration of 3450±10 m/s^2^ and took 6 ms to complete a jump, yet the main acceleration occurred in approximately 1 ms, peaking at 8650±10 m/s^2^ at the end of the whole jumping process. This scenario differs from that seen in other jumping insects, which have either a near-constant (Bonsignori et al. 2013) or gently increasing (Sutton and Burrows 2011) acceleration during take-off.

An individual *P.punctifrons* has an average mass of 1.6 mg, with the hind legs comprising only 17% of the total body mass. The jump pushes individuals to a final velocity of 5.58±0.5 m/s. The peak instantaneous power output (per unit mass) calculated for the hind legs in this species was 2.2±0.1 × 10^5^ W/kg, which is approximately 449 times that of the fastest-known muscle (Weis-Fogh and Alexander 1977) and some 100–200 times that of a powerful rally car engine.

In the field, flea beetles conduct contiguous jumps when encountering interference (based on our field observations in this study). When stimulated continuously in the laboratory (tested in this study), they can jump more than 30 times in a row without significant fatigue. Given that the power output of a catapult can be greater than the power input (Bennet-Clark and Lucey 1967; Alexander and Bennet-Clark 1977), because of the amplification of the power output, flea beetles thrust themselves across a distance hundreds of times their own body length in only a few milliseconds. In this way, flea beetles achieve extremely efficient and effective locomotion.

### Design of a bionic jumping leg (Fig. 6)

**Figure 6.**
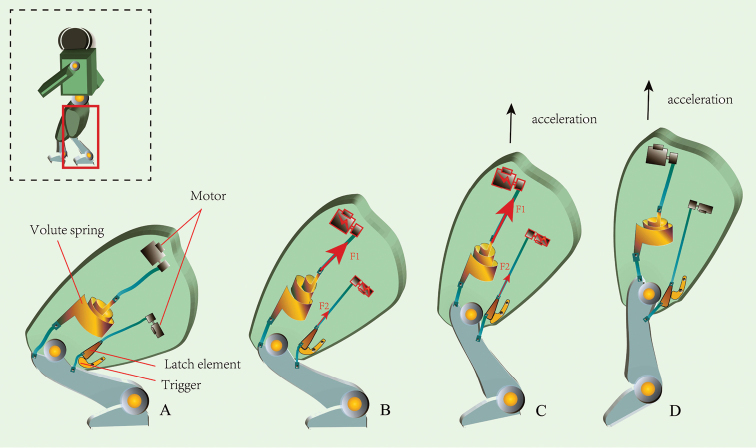
Bionic design of a jumping limb inspired by the flea beetle leg. **A** preparation position for jumping **B** building up elastic strain energy **C** triggering the jump **D** acceleration. The required energy for a jump is provided by two ‘motors’ operating simultaneously and generating the forces *F_1_* and *F_2_*, respectively. At the beginning (inset B), *F_1_* > *F_2_*, the ‘tibia’ rotates clockwise, but the ‘trigger’ blocks the process and leads to the stretching of ‘volute spring’. Thus, the work generated by the ‘motors’ is stored in the ‘volute spring’. At a certain stage (inset C), when the ‘trigger’ can no longer constrain the huge tension built up inside the femur, the ‘latch element’ dislodges suddenly from the ‘trigger’ and the huge amount of energy stored in the ‘volute spring’ is released. This leads to the explosive clockwise movement of the tibia. The leg thereby propels the robot into the air in an explosive manner. The leg can be switched to regular walking mode as required by operating only one ‘motor’ (one to flex the leg and the another to extend it) at a time.

Jumping can be a very effective mode of locomotion for small robots (Kovač et al. 2008). If designed using a catapult mechanism, a jumping leg could propel a robot into the air in an explosive manner (Fig. 6), while the robot could also return to a regular walking mode at any time by the catapult mechanism being switched off, such as a flea beetle does. A preliminary design of a robotic jumping leg is given in Figure 6. Its working principles are as follows. The required energy for a jump is provided by two ‘motors’ operating simultaneously and generating the forces *F_1_* and *F_2_*, respectively, *F_1_* is designed to be much greater than *F_2_*. At the beginning of a jump (Fig. 6B), when the motors are turned on, *F_1_* > *F_2_*, the ‘tibia’ starts to extend. However, a ‘trigger’ (mimicking the ‘elastic plate’ of the flea beetle leg) is designed to block the process and it leads to the stretching of a ‘volute spring’ (mimicking the metafemoral spring of the flea beetle leg). Thus, the work generated by the ‘motors’ is stored in the ‘volute spring’. At a certain stage (Fig. 6C), when the ‘trigger’ can no longer constrain the huge tension built up inside the femur, the ‘latch element’ (mimicking the triangular plate of the flea beetle leg) dislodges suddenly from the ‘trigger’ and the huge amount of energy stored in the ‘volute spring’ is released. This leads to the explosive extending movement of the tibia. The leg thereby propels the robot into the air.

The leg can also be switched to a regular walking mode as required by operating only one ‘motor’ at a time (as the ‘trigger’ will stop working when only one motor is turned on). In the regular walking mode, one motor could be turned on to flex the leg and the other to extend it, respectively.

## Discussion

Based on our findings, Barth’s (1954) theory of the flea beetle jumping mechanism is undoubtedly erroneous. Nadein and Betz (2016) correctly understood jumping processes of flea beetles. They reveal that the explosive catapult of flea beetle jump is established by the triangular plate being pressed against the inner wall of the distal femur. We revealed that the elastic plate can serve to prevent the triangular plate and inner wall of femur from injury due to the friction built up the jump, which in the end ensures much more explosive jumps.

The ‘elastic plate’ is not present in other jumping insects other than flea beetles. However, the locust has a similar structure called ‘Heitler’s lump’ (Bennet-Clark 1975). The ‘Heitler’s lump’ is a raised distal-ventral area of the inner wall of the hind leg with a slippery surface, which plays an important role in the lock system that helps to establish the catapult mechanism (Heitler 1974). Unlike the ‘Heitler’s lump’ which is as heavily sclerotized as the wall of the femur (Heitler 1974), the ‘elastic plate’ is only sclerotized at its base and has great elasticity at its middle and apex.

The mechanism of generating elastic potential energy in the flea beetle leg is like that in many other rapid-moving arthropods (for instance, flea, trap-jaw ants, mantis shrimp, etc.), which requires the ‘co-contraction’ process (extensor and flexor muscles contracting simultaneously). Flea beetles have evolved an enormous independent spring to aid the storage of elastic potential energy. This is significantly different from many other rapid-moving arthropods which usually only rely on exoskeleton or modified exoskeleton to store elastic potential energy, for instance, the semi-lunar process on distal part of hind femur in locusts (Burrows and Sutton 2012), the exoskeleton of the head in the trap-jaw ant (*Myrmoteras* sp., Larabee et al. 2017), the ‘saddle’ on the raptorial appendages in mantis shrimp (Patek et al. 2013; Patek 2015), etc. Moreover, instead of trigger muscles or latches employed in some other insects (Gronenberg 1996; Roberts and Azizi 2011), flea beetles utilize the elastic plate and triangular plate to control the timing of the instantaneous discharge of a catapult-like jump.

Some arthropod groups (see Table 1: mantis shrimps, snapping termite soldiers, trap-jaw spiders, etc.) have much greater acceleration or higher power output (per unit mass) than flea beetles. They usually strike only a part of the body to prey or defend. Unlike these arthropods, flea beetles need to propel the entire body to perform jumps, which results in the work being performed over a longer period of time and lower power output (Larabee et al. 2017).

There were several different modes of locomotion designed in bionic robots previously, such as: flying (Shyy et al. 1999), walking or trotting (Li et al. 2014), swimming (Cai et al. 2009), water walking (Suhr et al. 2005; Song and Sitti 2007) etc. Apart from these, jumping is another direction in robotic design, for instance, kangaroo inspired robot (Liu et al. 2014), bionic frog robot (Wang et al. 2008), bionic insect robot (Scarfogliero et al. 2007; Zaitsev et al. 2015), etc. In order to amplify the power output and achieve better locomotion efficiency, application of an elastic element seems to be inevitable. In our study, an elastic volute spring imitating the flea beetle metafemoral spring is employed to store and release elastic potential energy, which resembles some other designs in robotics (Scarfogliero et al. 2007; Kovač et al. 2008, 2010; Zaitsev et al. 2015). Besides the elastic potential energy design, the unique catapult mechanism and delicate structures inside the flea beetle leg were imitated and introduced into our design.

In addition to robotics, designs based on the flea beetle hind leg may be of use in other areas, such as engineering and industrial installations, in which the catapult mechanism and elastic elements could be crucially important.

**Table 1.** A comparison of characteristics between flea beetle jump and rapid movements in other arthropods.

**Arthropod species**	**Acceleration duration** (Appr.) · ms	**Velocity** (Appr.) · m/s	**Acceleration** (Appr.) · m/s^2^	**Power output (per unit mass)** (Appr.) · W/kg	**Movement type**
Flea beetles	1.1–7.7	0.7–5.6 (max. velocity)	100–3450	2.2×10^5^ (*Psylliodespunctifrons*)	Jumping
Fleas	1–2	0.8–1.9 (max. velocity)	960–1600	6×10^3^–1.4×10^4^	Jumping
Froghoppers	1–1.5	2.5–4.7 (max. velocity)	1667–5400	Unknown	Jumping
Locusts	20	2.2–3.1 (max. velocity)	100	450	Jumping
Termite soldiers	0.025	56 (average velocity)	Unknown	1.1×10^7^	Movement of mandibles
Trap-jaw ants	0.1–0.6	17–64 (max. velocity)	1×10^5^ –1×10^6^	2×10^4^– 3×10^5^	Movement of mandibles
Trap-jaw spiders	0.12	8.5 (average velocity)	Unknown	6.6×10^4^	Movement of chelicerae
Mantis shrimps	1–6	23–31 (max. velocity)	1×10^3^–1.5×10^5^	Unknown	Movement of raptorial appendages

*The data in Table 1 are cited from the following studies: flea beetles (Brackenbury and Wang 1995; Nadein and Betz 2016; present study), fleas (Bennet-Clark and Lucey 1967; Sutton and Burrows 2011), froghoppers (Burrows 2006), locusts (Heitler 1974; Bennet-Clark 1975), termite soldiers (Seid et al. 2008), trap-jaw spiders (Wood et al. 2016), trap-jaw ants (Patek et al. 2006; Larabee et al. 2017), Mantis shrimps (Patek et al. 2004; Patek 2015).

## Supplementary materials

Supplementary material 1 Movie S1. A model of the catapult mechanism in the hind legs of flea beetles Data type: multimedia https://binary.pensoft.net/file/383074This dataset is made available under the Open Database License (http://opendatacommons.org/licenses/odbl/1.0/). The Open Database License (ODbL) is a license agreement intended to allow users to freely share, modify, and use this Dataset while maintaining this same freedom for others, provided that the original source and author(s) are credited.Yongying Ruan, Alexander S. Konstantinov, Guanya Shi, Yi Tao, You Li, Andrew J. Johnson, Xiaozhu Luo, Xinying Zhang, Mengna Zhang, Jianing Wu, Wenzhu Li, Siqin Ge, Xingke Yang

Supplementary material 2 Movie S2. The take-off strategy of *Asiophridaxanthospilota* Baly as filmed by a high-speed camera Data type: multimedia https://binary.pensoft.net/file/383075This dataset is made available under the Open Database License (http://opendatacommons.org/licenses/odbl/1.0/). The Open Database License (ODbL) is a license agreement intended to allow users to freely share, modify, and use this Dataset while maintaining this same freedom for others, provided that the original source and author(s) are credited.Yongying Ruan, Alexander S. Konstantinov, Guanya Shi, Yi Tao, You Li, Andrew J. Johnson, Xiaozhu Luo, Xinying Zhang, Mengna Zhang, Jianing Wu, Wenzhu Li, Siqin Ge, Xingke Yang

Supplementary material 3 Movie S3. Internal structures of the hind leg of *Asiophridaxanthospilota* Baly Data type: multimedia https://binary.pensoft.net/file/383076This dataset is made available under the Open Database License (http://opendatacommons.org/licenses/odbl/1.0/). The Open Database License (ODbL) is a license agreement intended to allow users to freely share, modify, and use this Dataset while maintaining this same freedom for others, provided that the original source and author(s) are credited.Yongying Ruan, Alexander S. Konstantinov, Guanya Shi, Yi Tao, You Li, Andrew J. Johnson, Xiaozhu Luo, Xinying Zhang, Mengna Zhang, Jianing Wu, Wenzhu Li, Siqin Ge, Xingke Yang

Supplementary material 4 Movie S4. Test and confirmation of the catapult mechanism in the flea beetle hind leg Data type: multimedia https://binary.pensoft.net/file/383077This dataset is made available under the Open Database License (http://opendatacommons.org/licenses/odbl/1.0/). The Open Database License (ODbL) is a license agreement intended to allow users to freely share, modify, and use this Dataset while maintaining this same freedom for others, provided that the original source and author(s) are credited.Yongying Ruan, Alexander S. Konstantinov, Guanya Shi, Yi Tao, You Li, Andrew J. Johnson, Xiaozhu Luo, Xinying Zhang, Mengna Zhang, Jianing Wu, Wenzhu Li, Siqin Ge, Xingke Yang
